# Reviews

**Published:** 1884-04

**Authors:** 


### Reviews.

INSANITY AND ITS TREATMENT. By SAMUEL WORCESTER, M.D. New York: Bœricke & Tafel. 1882. Cloth, $5.

INSANITY: Its Classification, Diagnosis, and Treatment. By E. C. SPITZKA, M.D. New York: Bermingham & Co. 1883. Cloth, $3.

A TREATISE ON INSANITY IN ITS MEDICAL RELATIONS. By WILLIAM A. HAMMOND, M.D. New York: D. Appleton & Co. 1883. Cloth, $5.

PSYCHOLOGICAL MEDICINE. By E. C. MANN, M.D. Philadelphia: P. Blakiston & Co. 1883. Cloth, $5.

MEDICAL JURISPRUDENCE. By A. MC. L. HAMILTON, M.D. New York: Bermingham & Co. 1884. Cloth, $5.

The progress and features of American psychiatry in the last few years have been decidedly flattering to nativistic physicians, and the works just enumerated are striking illustrations of the various phases of thought which are to be detected in the medical world on this subject. The first work is a very fair condensation of the older American and more recent European literature. It is written by a homœopathic renegade from the regular profession, and the only original feature about the book is the decidedly original ideas as to the purpose of therapeusis. Chloral hydrate is recommended in thirty grain doses, and other drugs in equally large doses; in case homœopathic therapeusis fails, for the purpose of preventing "the patient passing into the hands of the old school profession." If the therapeutic vagaries were removed, the work would be of no little value.

The second work is by a very well known physician. It is up to the times. It is well written, but cannot be skimmed; it must be studied. The classification adopted is modified from that of Krafft-Ebing and in the opinion of Tamborini, one of the leading Italian alienists, (no mean authority), is a decided improvement on that classification. Like everything scientific, it is not simple, but requires careful study. American alienists are quoted at length. While the author has thus done unusual justice to the alienists of his native land, he has not neglected those of Europe. A perhaps objectionable feature is the severely critical analysis to which the author subjects the writings of persons made alienists by the grace of politicians. Reference is made to the much neglected question of simulation of insanity, and concealment of insanity by the insane. The classification of the author is as follows, divesting it of its generic and specific relations: "Mania, melancholia, katatonia, transitory frenzy, stuporous insanity, primary and secondary confusional insanity, primary deterioration, terminal dementia, senile dementia, hebephrenia, paretic dementia, luetic dementia, dementia from coarse brain disease, delirium grave, alcoholic, hysterical, epileptic and periodical insanities, idiocy, imbecility, cretinism, monomania, traumatic, choreic, post febrile, rheumatic, gouty, phthisical, sympathetic and pellagrous insanities." It will be obvious that this is well adapted for clinical purposes. The book is a sound, practical, scientific guide in the study of psychiatry.

Dr. Hammond's work makes an attempt to demarcate between legal and medical insanity. The best legal minds have concluded that what is fact in science cannot be fiction in law; and, viewed from this standpoint, Dr. Hammond's views are, as has been said by the *Alienist and Neurologist*, a weak compromise with error. The classification of Dr. Hammond is complex, and, it must be confessed, inconsistent. It is as follows: 1. Perceptional insanities, in which there are derangements of one or more of the perceptions. A, illusions; B, hallucinations. 2. Intellectual insanities, in which the chief manifestations of mental disorder relate to the intellectual being of the nature of false conceptions, or clearly abnormal conceptions. A, intellectual monomania with exaltation; B, intellectual monomania with depression; C, chronic intellectual mania; D, reasoning mania; E, intellectual subjective morbid impulses; F, intellectual objective morbid impulses. 3. Emotional insanities, in which the mental derangement is chiefly exhibited with regard to the emotions. A, emotional monomania; B, emotional morbid impulse; C, simple melancholia; D, melancholia with delirium; E, melancholia with stupor; F, hypochondriacal mania or melancholia; G, hysterical mania; H, epidemic insanity. 4. Volitional insanities, which are characterized by derangement of the will, either by its abnormal predominance or inertia. A, volitional morbid impulse; B, aboulomania (paralysis of the will). 5. Compound insanities, in which two or more categories of mental faculties are markedly involved. A, acute mania; B, periodical insanity; C, hebephrenia; D, circular insanity; E, katatonia; F, primary dementia; G, secondary dementia; H, senile dementia; I, general paralysis. 6. Constitutional insanities, which are the result of a preexisting physiological or pathological condition, or of some specific morbid condition affecting the system. A, epileptic insanity; B, puerperal insanity; C, pellagrous insanity; D, choreic insanity, etc. 7. Arrests of mental development. A, idiocy; B, cinism. It is quite unnecessary to do more than quote this classification, to show that it is somewhat inconsistent. The classification is a stumbling block, rather than an aid. The descriptions of the generally accepted forms are models of excellence. The work, as a whole, can be recommended as a good guide in clinical psychiatry, but contains unsound and dangerous opinions on legal medicine, as it enunciates doctrines opposed, as even its author admits, to abstract justice.

The first thing about Dr. Mann's book which strikes the reader, is its illy digested character. It looks as if it had been "scissored" from the author's contributions to periodical literature. It is, when carefully sifted, a good guide in clinical psychiatry; a poor one in pathological histology, since the changes produced by manipulation are not guarded against. The work enunciates sound views in legal medicine. Carefully edited and skilfully condensed, it could be made a good text-book. Its chief faults are that it lacks clearness and system.

The last work has all the faults of Dr. Mann's book, and none of its virtues. Its author is not acquainted with the fact that the insane feign insanity. He is unacquainted with many more facts an alienist ought to know. The book is worthy of the author, who has declared upon the witness stand that "if the insane knew right from wrong, and *could* not control themselves, they should be punished the same as the sane."

Three of these woıks are desirable acquisitions. Spitz kas book requires brain work in its perusal, but, its general principles grasped, proves of great value. Hammond's work is easily perused, readily assimilated, but is not a very safe guide in practice, because of the exceptional views of its author. Dr. Mann's book, from its lack of system and prolixity, requires equal labor in its perusal with that of Dr. Spitzka, and this labor is not as well rewarded. Worcester's book is valueless because of its *dishonest* therapeutic views, while Hamilton's book has all the vices of the works of Hammond and Mann in a greater degree than either, and none of their virtues, and displays the erratic views of an author who proclaims that a *low* facial angle is a sign of intellectuality. JAS. G. KIERNAN.

---

THE HOT WATER CURE.—A writer in the *Lancet* (Dec. 29) gives his experiencc of hot water as a curative agent in indigestion, for whlch he has suffered for many years, and by tho judicious use of which he has been relieved. He says half to a teacupful of hot water (not boiling nor yet lukewarm) sipped before a meal will enable nine out of every ten sufferers from indigestion to digest that meal with comfort; and in cases where a fulness and uncomfortableness are felt after a meal, a similar quantity taken in a similar manner will give relief.—*Maryland Medical Journal.*

THE names of two distinguished Western physicians are in scribed to-day on the long roll of the majority, Dr. Lunsford P. Yandell, of Louisville, and Dr. George Englemann, of St. Louis. Each of these gentlemen had won a brilliant reputation in the profession; and each had an authority that was respected, not only in this country, but abroad. We can ill afford to lose the lustre of these names from the list of eminent professional men in the West.

